# Highly recruited brown adipose tissue does not in itself protect against obesity

**DOI:** 10.1016/j.molmet.2023.101782

**Published:** 2023-07-25

**Authors:** Gabriella von Essen, Erik Lindsund, Elaina M. Maldonado, Petr Zouhar, Barbara Cannon, Jan Nedergaard

**Affiliations:** 1 Department of Molecular Biosciences, The Wenner-Gren Institute, The Arrhenius Laboratories F3, Stockholm University, SE-106 91 Stockholm, Sweden; 2 Laboratory of Adipose Tissue Biology, Institute of Physiology of the Czech Academy of Sciences, Videnska 1083, CZ-142 00 Prague, Czech Republic

**Keywords:** Diet-induced thermogenesis, UCP1, Body weight regulation, Beige adipose tissue, Adipostat, Glucose homeostasis

## Abstract

**Objective:**

The possibility to counteract the development of obesity in humans by recruiting brown or brite/beige adipose tissue (and thus UCP1) has attracted much attention. Here we examine if a diet that can activate diet-induced thermogenesis can exploit pre-enhanced amounts of UCP1 to counteract the development of diet-induced obesity.

**Methods:**

To investigate the anti-obesity significance of highly augmented amounts of UCP1 for control of body energy reserves, we physiologically increased total UCP1 amounts by recruitment of brown and brite/beige tissues in mice. We then examined the influence of the augmented UCP1 levels on metabolic parameters when the mice were exposed to a high-fat/high-sucrose diet under thermoneutral conditions.

**Results:**

The total UCP1 levels achieved were about 50-fold higher in recruited than in non-recruited mice. Contrary to underlying expectations, in the mice with highly recruited UCP1 and exposed to a high-fat/high-sucrose diet the thermogenic capacity of this UCP1 was completely inactivate. The mice even transiently (in an adipostat-like manner) demonstrated a *higher* metabolic efficiency and fat gain than did non-recruited mice. This was accomplished without altering energy expenditure or food absorption efficiency. The metabolic efficiency here was indistinguishable from that of mice totally devoid of UCP1.

**Conclusions:**

Although UCP1 protein may be available, it is not inevitably utilized for diet-induced thermogenesis. Thus, although attempts to recruit UCP1 in humans may become successful as such, it is only if constant activation of the UCP1 is also achieved that amelioration of obesity development could be attained.

## Highlights

• Enhanced amounts of UCP1 are expected to counteract obesity.
• However, pre-enhanced UCP1 is not activated to bring about augmented diet-induced thermogenesis (DIT).
• On the contrary, metabolic efficiency may be increased to expand energy stores.
• This is accomplished without decreasing energy expenditure or food absorption efficiency.
• Not only UCP1 augmentation but also UCP1 activation is essential for enhanced DIT and protection from obesity.

## Graphical abstract

**Figure:**
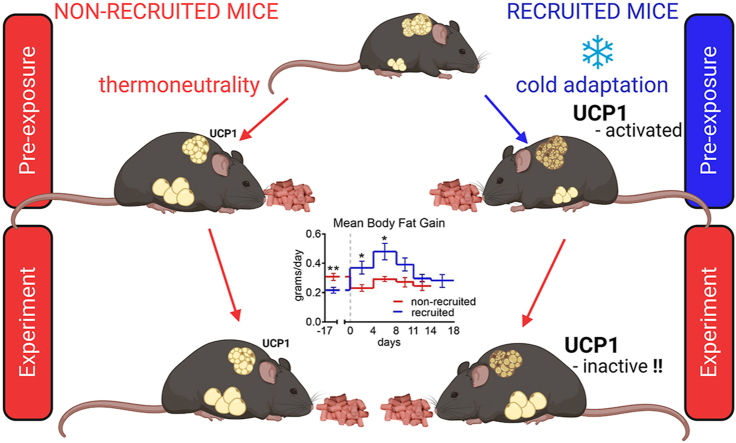

## 1 Introduction

Presently, much scientific and pharmaceutical effort is channeled into the identification and development of drugs, nutraceuticals and cellular pathways that augment the amounts of UCP1 in brown and brite/beige adipose tissues (“browning agents”) [[1], [2], [3], [4], [5], [6]]. The underlying tenet is that an augmented amount of uncoupling capacity in the form of increased amounts of UCP1 will inherently lead to an increase in the total organismal combustion of food and thus counteract the development of obesity – and in the best case even reduce obesity in those who have already been afflicted. Additionally, it is envisaged that an increased amount of UCP1 and of brown and brite/beige adipose tissues would manifest with metabolically positive effects, such as being a sink for glucose and fatty acids, leading to a lowering of plasma glucose levels and thus also, through this, to decreased metabolic risks [7].

However, in reality, there is a deficit of studies demonstrating the validity of what seems to be the underlying tenet: that augmented UCP1 amounts in themselves protect against obesity.

In the present study, we have utilized the most powerful browning agent presently known – i.e. chronic cold – to vastly augment the total amount of UCP1 in mice (here referred to as (thermogenically) “recruited mice”). We then aimed to quantify the ability of this high amount of UCP1 and the fully recruited brown and brite/beige adipose tissues to combust the excess calories encountered in a high-fat diet. Particularly, we utilized a diet that has earlier been demonstrated to lead to UCP1-dependent diet-induced thermogenesis, i.e. this diet in itself leads to an increase in the total amount of UCP1 and to an increased UCP1-dependent meal-induced metabolic rate [8]. This implies that the diet, directly or indirectly, can activate (sympathetic) pathways that stimulate the brown and/or brite/beige tissues and the UCP1 therein. The recruited mice exposed to this diet may thus be said to have regulatory access to a much higher thermogenic capacity than that found in non-recruited mice. This should enable the mice to powerfully oppose energy accumulation in the form of obesity.

We found, however, contrary to these tenets, that the high amounts of UCP1 were not exploited by the mice to counteract fattening. Rather, remarkably, the mice with highly recruited brown and brite/beige adipose tissue were able to increase their metabolic efficiency even *above* that of non-recruited mice, to further accelerate acquisition of extra body fat, despite possessing large amounts of UCP1. Thus, even the stimulus of a diet that is proven to be thermogenic does not automatically engage the thermogenic capacity of the UCP1 that is present.

## 2 Abridged materials and methods

For detailed methods descriptions, see Supplement.

### 2.1 Animals

All experiments were approved by the North Stockholm Animal Ethics Committee. Cohorts of wild-type male C57Bl/6 mice (8–12 weeks old) were exposed to a light–dark 12/12 h cycle, were single-cage and had *ad libitum* access to a high-fat diet (D12451, Research diets) and water. Before the studies started, the mice were kept at 22 °C. Basically, in all experiments, half of the mice were pre-exposed for 33 days to cold in order to induce very high recruitment of their brown and brite/beige adipose tissues (the “recruited” mice); the other half was placed at principally thermoneutral temperatures (the “non-recruited” mice) (29 °C). The experiments were started by transferring the recruited mice to thermoneutrality while keeping the high-fat diet. Body fat content and lean body mass of mice were measured by *in vivo* magnetic resonance imaging (MRI), and cohorts of mice were killed after the indicated number of days and their tissues further examined.

Oxygen consumption and carbon dioxide production were measured by indirect calorimetry (INCA Systems) at 30 °C, and glucose, insulin and pyruvate tolerance tests were performed with routine methods, injecting the agents in proportion to lean body weight. Feces energy content was determined by scanning microcalorimetry.

### 2.2 Protein analysis

**Figure 1:**
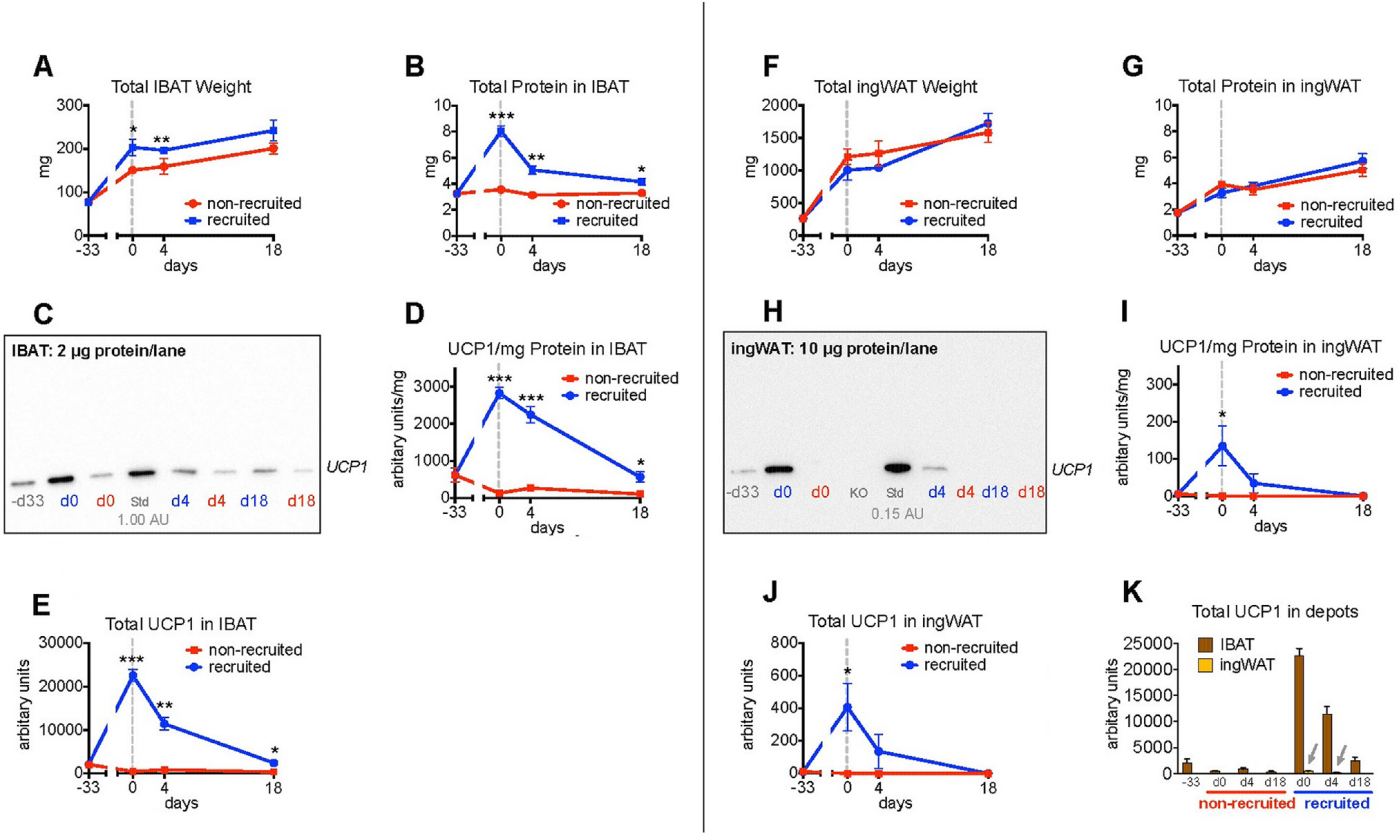
**Large increase in total UCP1 protein amounts after recruitment**. Adult mice (35 male C57Bl/6) were given high-fat diet and placed at two ambient temperatures (“non-recruited” at 29 °C and “recruited” at 4 °C) on day −33. At day 0, the recruited mice were transferred to 29 °C (thermoneutrality). At the indicated time-points, 5 mice from each group were euthanized and interscapular brown adipose tissue and inguinal white adipose tissue were excised, weighed and analyzed. **A:** Wet weight of IBAT. **B:** Total protein content in IBAT. **C:** Representative Western blot of UCP1 protein levels in IBAT. Non-recruited shown with red letters and recruited shown with blue letters, d0 = day 0 etc. The standard (Std) is here set to 1.00 AU (arbitrary units) of UCP1, as indicated. All other lanes were loaded with 2 μg protein per sample. **D:** UCP1 protein per mg protein. **E:** Total amount of UCP1 in IBAT, calculated as values from B multiplied with corresponding values from D. **F:** Wet weight of ingWAT. **G:** Total protein content in ingWAT. **H:** Representative Western blot of UCP1 protein levels in ingWAT. The standard (Std) loaded in this case was 0.15 AU (arbitrary units) UCP1 (compared to 1.00 AU UCP1 in the Western blots analyzing IBAT). The other lanes were loaded with 10 μg protein per sample, including a sample from a UCP1(−/−) mouse (KO). **I:** UCP1 protein per mg protein. **J:** Total amount of UCP1 in ingWAT, obtained as values from G multiplied with corresponding values from I. **K:** Comparison of total amount of UCP1 in IBAT and ingWAT. Arrows point to the only measurable ingWAT data obtained. A, B, D–G, I–K: n = 5. Statistically significant differences indicated when p-values ≤0.05 as ∗ for recruited versus non-recruited; ∗∗ for p ≤ 0.01 and ∗∗∗ for p ≤ 0.001. IBAT = interscapular brown adipose tissue; ingWAT = inguinal white adipose tissue; UCP1 = uncoupling protein 1. (For interpretation of the references to colour in this figure legend, the reader is referred to the Web version of this article.)

The entire depots of interscapular brown adipose tissue and inguinal white adipose tissue were dissected out and weighed. The tissues were immediately frozen in liquid nitrogen and stored at −80 °C. For protein analysis, tissue was homogenized in RIPA buffer. For determination of UCP1 protein levels, 2 or 10 μg protein (as detailed in legend to Figure 1) were loaded on an SDS-polyacrylamide gel. The amount of UCP1 in a “standard” brown-fat sample was set to 1.00 AU. UCP1 protein was determined by incubation with UCP1 polyclonal antibodies (prepared in rabbit from the C-terminal decapeptide of mouse UCP1) as primary antibody and Anti-Rabbit IgG HRP-linked antibody (Cell Signaling) as secondary antibody. Dilution of both antibodies was 1:12,000.

## 3 Results

The purpose of this investigation was to examine whether highly recruited brown and brite/beige adipose tissues protect against diet-induced obesity and in general improve metabolic parameters. In order to obtain very high amounts of UCP1, mice were therefore pre-acclimated to cold (“recruited mice”). Thereafter, they were transferred to thermoneutral conditions. All mice were exposed to a diet that in itself promotes diet-induced thermogenesis and a modest increase in total UCP1 amounts [8]. We investigated the ability of the exceptionally high amounts of UCP1 that were induced by cold acclimation to protect the mice against the expected obesity when they were exposed to the thermogenesis- and obesity-inducing diet.

### 3.1 Large increase in total UCP1 protein amount in IBAT caused by the recruitment

To ascertain that the experimental set-up profoundly recruited brown adipose tissue, the potential thermogenic capacity of the tissue was estimated (Figure 1), both during the recruitment phase (d -33 to d 0) and during the actual experimental phase (d 0 to d 18). The data from the mice that were induced to possess highly recruited brown adipose tissue are shown with blue lines/letters in the figures. The wet weight of brown adipose tissue was doubled during the recruitment process (Figure 1A), and the total amount of protein in the tissue was more than doubled (Figure 1B). That the BAT was highly recruited was particularly apparent on the representative western blot for UCP1, showing a clear increase in UCP1 content per μg protein (Figure 1C, d0 versus d -33). Compilations of data from all the mice emphasized the much higher content of UCP1 per μg tissue protein for the thermogenically recruited mice on day 0 versus that in the non-recruited mice (i.e. the mice that had been kept at thermoneutrality (Figure 1D)). Also during the experimental phase when both groups were at thermoneutrality, the UCP1 level in the recruited mice remained higher than in the non-recruited, even after 18 days (Figure 1D).

The physiologically important parameter is, however, not the relative UCP1 content but the total amount of UCP1 in the IBAT depot (i.e. level of UCP1/μg protein multiplied with total protein; Figure 1D data multiplied with Figure 1B data) [9,10]. Immediately after the end of the recruitment process, i.e. day 0, the UCP1 amount was 50 times higher in the recruited brown adipose tissue than in the non-recruited (Figure 1E). Following transfer to thermoneutrality, the total amount of UCP1 decreased, but on day 18, it was still 7 times higher than in the non-recruited state; thus, a significantly elevated potential capacity for thermogenesis remained in the recruited mice. The physiological half-life of total UCP1 during de-recruitment (calculation not shown) was approximately 6 days, which is in agreement with earlier in-vivo observations [11,12].

In contrast, in the mice that were maintained constantly at thermoneutrality, little happened (red curves in Figure 1) with respect to brown adipose tissue wet weight and total protein content (Figure 1A,B). Since the mice had been at 22 °C (below the thermoneutral zone) before the experiment and were transferred to thermoneutrality on d −33, the necessity for nonshivering thermogenesis was removed, and thus the UCP1 amounts decreased in these mice during the acclimation period (Figure 1C,D,E).

### 3.2 Despite a large *relative* increase of UCP1 in inguinal WAT after cold-induced recruitment, total UCP1 amounts in inguinal WAT are negligible

Certain white adipose tissue depots possess the ability to become thermogenic (i.e. to express UCP1 and incorporate it functionally into mitochondria), referred to as browning or to become brite or beige [2,[13], [14], [15], [16], [17]]. It has even been discussed that such brite/beige depots could be particularly significant for counteracting obesity through increased thermogenesis [17,18]. Inguinal white adipose tissue (ingWAT) is the white adipose depot that most potently undergoes browning. Hence, we examined the amount of UCP1 in the inguinal depot after cold-induced recruitment.

IngWAT wet weight did not differ at any time point between the mice that were recruited or non-recruited (Figure 1F); in both groups, ingWAT increased 6-fold during the 7 weeks of the experiment. The total protein content in ingWAT increased modestly with time and did not differ between the groups at any time point (Figure 1G).

In the cold-acclimated mice, the Western blot demonstrated a large increase in UCP1 levels per μg protein during the recruitment phase from day −33 to day 0 (Figure 1H). However, after 18 days at thermoneutrality, UCP1 protein in the recruited ingWAT was undetectable, just as it was in all non-recruited ingWAT samples (Figure 1HI). Thus, at the end of the recruitment phase, on day 0, ingWAT demonstrated a very high browning level, relative to the level in non-recruited mice (Figure 1I). However, when the total amount of UCP1 protein in the ingWAT was calculated (Figure 1J), it became evident that the amount was some 50 times lower than that in IBAT. Consequently, the total amount of UCP1 in ingWAT, despite the large increase, remained virtually negligible compared to the amount in IBAT, even after full recruitment (Figure 1K; ingWAT levels indicated by arrows).

### 3.3 No *innate* obesity protection even in the presence of high amounts of UCP1

We demonstrated above (sections 3.1, 3.2) that the total thermogenic capacity (estimated as the total amount of UCP1) was dramatically increased by the end of the recruitment process. The main aim of the present investigation was thus to establish to what extent this very large amount of UCP1 and recruited BAT could counteract the development of obesity that is expected to occur in mice fed a high-fat-diet and housed at a thermoneutral temperature. In other words, will this extra thermogenic capacity in the recruited mice be called into use to protect against obesity when the mice are exposed to the obesity-inducing but also thermogenesis-inducing high-fat diet [8].

**Figure 2:**
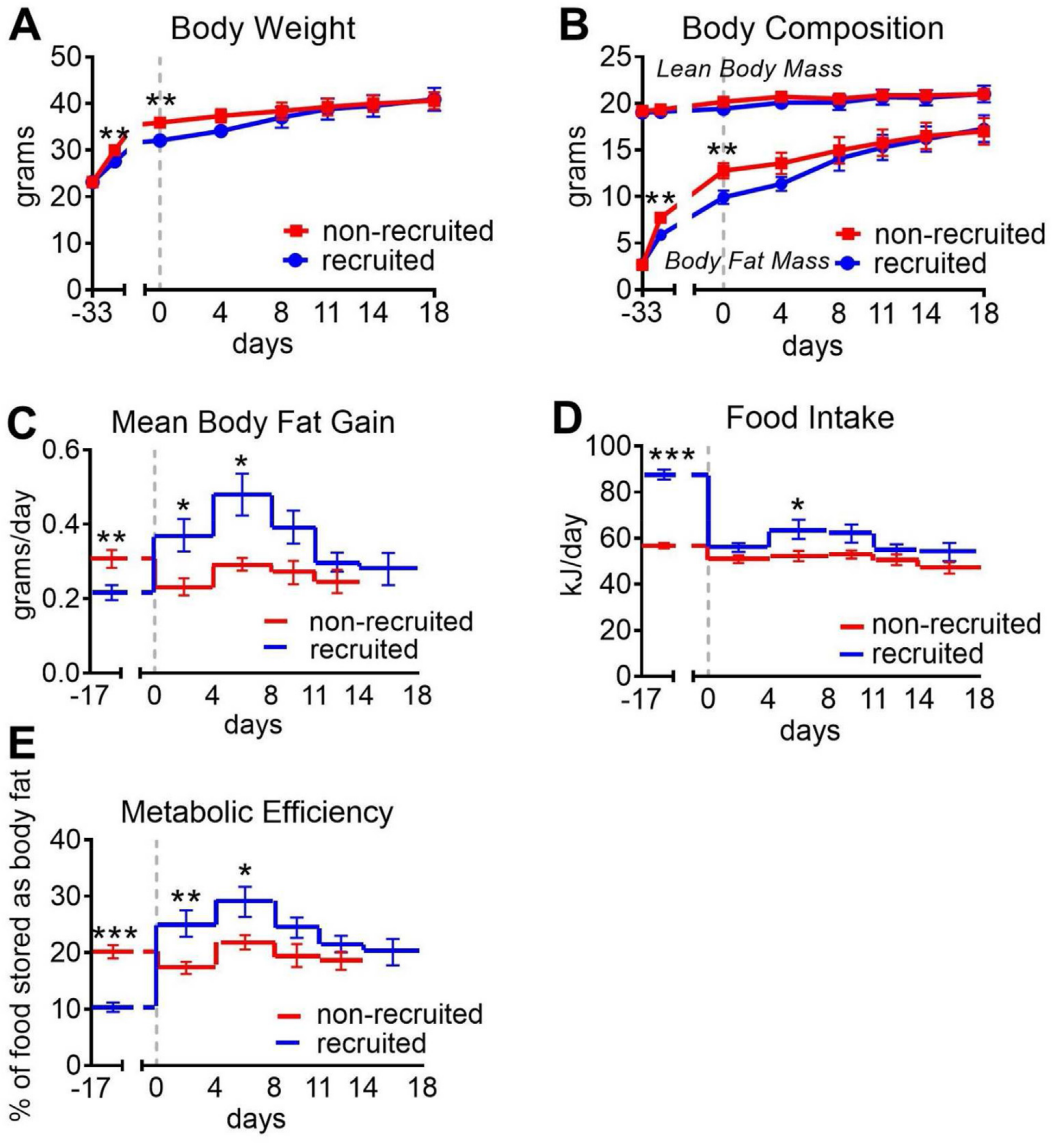
**No obesity protection despite high UCP1 amounts**. The mice from Figure 1 were monitored. Body composition was measured on the days indicated and food intake was measured from day −17 to day 18. Initial weights at day −33 were 23.2 ± 0.3 for non-recruited mice and 23.2 ± 0.4 for recruited mice (means ± SE). **A:** Body weight. **B:** Body composition; lean body mass and body fat mass. **C:** Body fat gain per day at different time periods (day 18 for non-recruited not shown for technical reasons). **D:** Food intake. **E:** Metabolic efficiency expressed as % of the energy in the ingested food stored as body fat. n = 15 (day -33 – 0); n = 10 (day 4); n = 5 (day 8–18). Significances as described in legend to Figure 1.

At the end of the pre-phase (day 0), the non-recruited mice had a significantly higher body weight and possessed significantly more body fat than the recruited mice, but there was no difference in lean body mass (Figure 2A,B).

As seen in Figure 2C, during the entire experiment, the non-recruited mice gained 0.2–0.3 g body fat per day. The mice with recruited brown adipose – when transferred to thermoneutrality – could be expected to increase their obesity at a similar rate, were it not for the large amount of UCP1 that they had acquired. The prediction was thus that this overcapacity of thermogenic potential would be activated to perform augmented diet-induced thermogenesis. This should result in mice that were resistant to the increase in body weight, at least more resistant than the mice with unrecruited brown adipose tissue.

However, contrary to these expectations, when the mice with recruited brown adipose tissue were transferred to the thermoneutral temperature, the mice immediately began to accumulate an excess of fat. Particularly, as seen in Figure 2C, the daily increase in body fat in these mice rose to more than 0.4 g; indeed, during the first 11 days after the transfer, the mice accumulated about 170% of the amount of fat accumulated by the non-recruited mice. This occurred despite the 10–50 times higher amount of UCP1 in these mice, as compared to the non-recruited mice. There was thus no protection against obesity as a consequence of the large amount of UCP1, even though the mice were exposed to the thermogenesis-inducing high-fat diet [8].

After the first 11 days, the recruited mice gained fat at the same rate as the non-recruited mice. An explanation for the transience of this accelerated lipid accumulation may be deduced from the graph depicting the total fat amount in the two mouse groups. As seen (Figure 2B), when transferred to thermoneutrality, the mice with recruited brown adipose tissue had substantially less body fat than had the non-recruited. Apparently, the mice immediately strived to attain the same amount of body fat as the non-recruited mice and thus increased their rate of fat deposition. However, after 11 days, they had reached the same level of adiposity as the non-recruited mice. The mice then ceased the accelerated fat deposition, aligning themselves with the non-recruited mice.

### 3.4 Explanations for the increased lipid accumulation

The accelerated fat deposition could be due to increased food intake, decreased energy expenditure, and/or increased assimilation efficiency in the gut.

As seen in Figure 2D, non-recruited mice had a constant level of food intake during the entire study. During the pre-phase, the “recruited” mice had a 50% higher food intake compared to the non-recruited mice, as expected, due to the need to compensate for the high heat loss in the cold. When the recruited mice were transferred to thermoneutrality, they immediately reduced their food intake to a level similar to but slightly higher than the non-recruited group (Figure 2D).

In the mice remaining at thermoneutrality throughout the experiment, metabolic efficiency, calculated as the percentage of the total energy intake that becomes stored as body fat, was stable and about 20%. In the recruitment pre-phase, the recruiting mice had only half this efficiency (10%). However, when the recruited mice were transferred to thermoneutrality, they immediately increased their efficiency 3-fold to about 30% (Figure 2E), and the metabolic efficiency of the high-UCP1-containing mice thus markedly exceeded that of the non-recruited mice for almost 2 weeks.

### 3.5 No difference in energy expenditure after transfer

**Figure 3:**
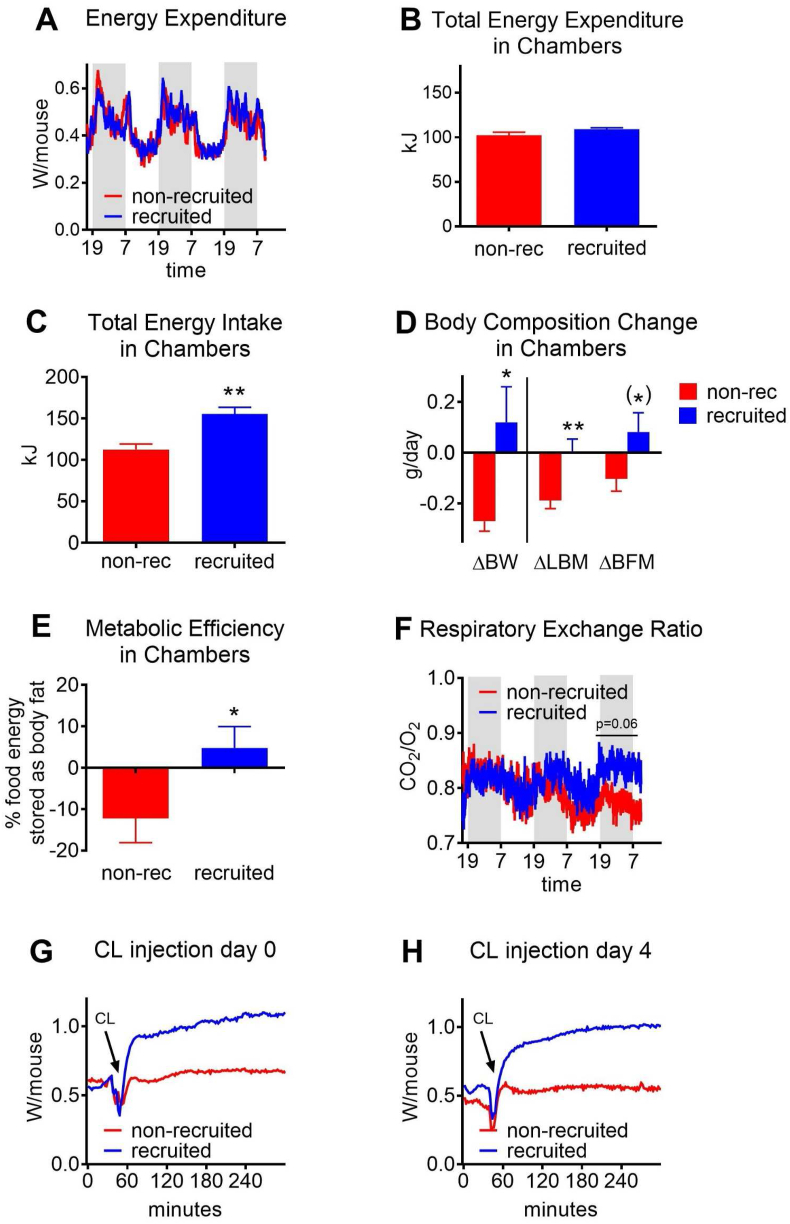
**No difference in energy expenditure after transfer to warm**. An independent group of mice were treated as the mice in Figure 1, Figure 2. At day 0, i.e. on transfer of recruited mice into thermoneutrality, the mice were placed for three nights and two days in metabolic chambers kept at 29 °C. Oxygen consumption and carbon dioxide production were measured. The first 3 h are not shown. Starting weights at day −33 were 24.3 g ± 0.3 for non-recruited and 24.8 g ± 0.5 for recruited (means ± SE). **A:** Energy expenditure, expressed as Watt per mouse. As the lean body weights of these mice were very similar (non-recruited: 20.0 ± 0.3 g; recruited 20.3 ± 0.3 g; means ± SE), the curves are unchanged if expressed per g lean body mass. **B:** Total energy expenditure while in the chambers (68 h). The data were further analysed by ANCOVA with body weight as additional variable (see also supplementary figure); this did not affect the statistical outcome. **C:** Total energy intake while in the chambers (68 h). The data were further analysed by ANCOVA with body weight as additional variable (see also supplementary figure); this did not affect the statistical outcome. **D:** Changes in body composition while in the chambers; ΔBW: change in body weight; ΔLBM: change in lean body mass; ΔBFM: change in body fat mass. **E:** Metabolic efficiency expressed as % of the energy in the food stored as body fat. **F:** Mean respiratory exchange ratio: V_CO2_/V_O2_. **G:** Effect of CL-316,243 injection on energy expenditure in mice immediately at the end of the recruitment period; mean values. **H:** As G, but performed in mice on day 4 after the end of the recruitment period. n = 6–7 for all panels. Significances as described in legend to Figure 1; (∗) indicates p ≤ 0.10.

The significantly increased total metabolic efficiency in the recruited versus non-recruited mice (Figure 2E) could imply a state of decreased energy expenditure, which could thus explain the high increase in body fat gain. To examine whether a lowered energy expenditure was induced, a group of mice with the same background, conditions and treatments as those described above were placed in calorimetric chambers for three days starting at day 0. This was thus the first day the recruited mice were exposed to thermoneutrality. Despite some 50-fold difference in UCP1 amount between the two groups, there was no detectable difference in energy expenditure between the groups (Figure 3A,B). However, also here, the recruited mice ate more (Figure 3C) and also here gained more weight, mainly body fat, while the non-recruited mice under these circumstances showed decreased body fat and lean body mass in the chambers (Figure 3D). (This weight loss did not happen in mice not exposed to the calorimetric chambers (Figure 2B), and it was thus probably a stress-response elicited by the change in environment.) Consequently, the recruited mice also here had higher metabolic efficiency compared to the non-recruited mice (Figure 3E). Even though the non-recruited mice lost body fat, the difference in absolute values between the two groups was basically the same as in experiment 1 for the first three days, a difference of some 0.2 g of body fat/day (Figure 3D).

The respiratory exchange ratio (RER) indicates which substrate is being utilized. The theoretical RER of the high-fat diet used here is 0.84 (see Supplementary Methods). As seen in Figure 3F, the non-recruited mice combusted relatively more lipids than did the recruited mice after one day in the chambers, in agreement with them utilizing their fat depots (Figure 3D).

These studies thus indicated that the increased metabolic efficiency was not due to a lowered energy expenditure.

### 3.6 No change in efficiency of food energy uptake

**Figure 4:**
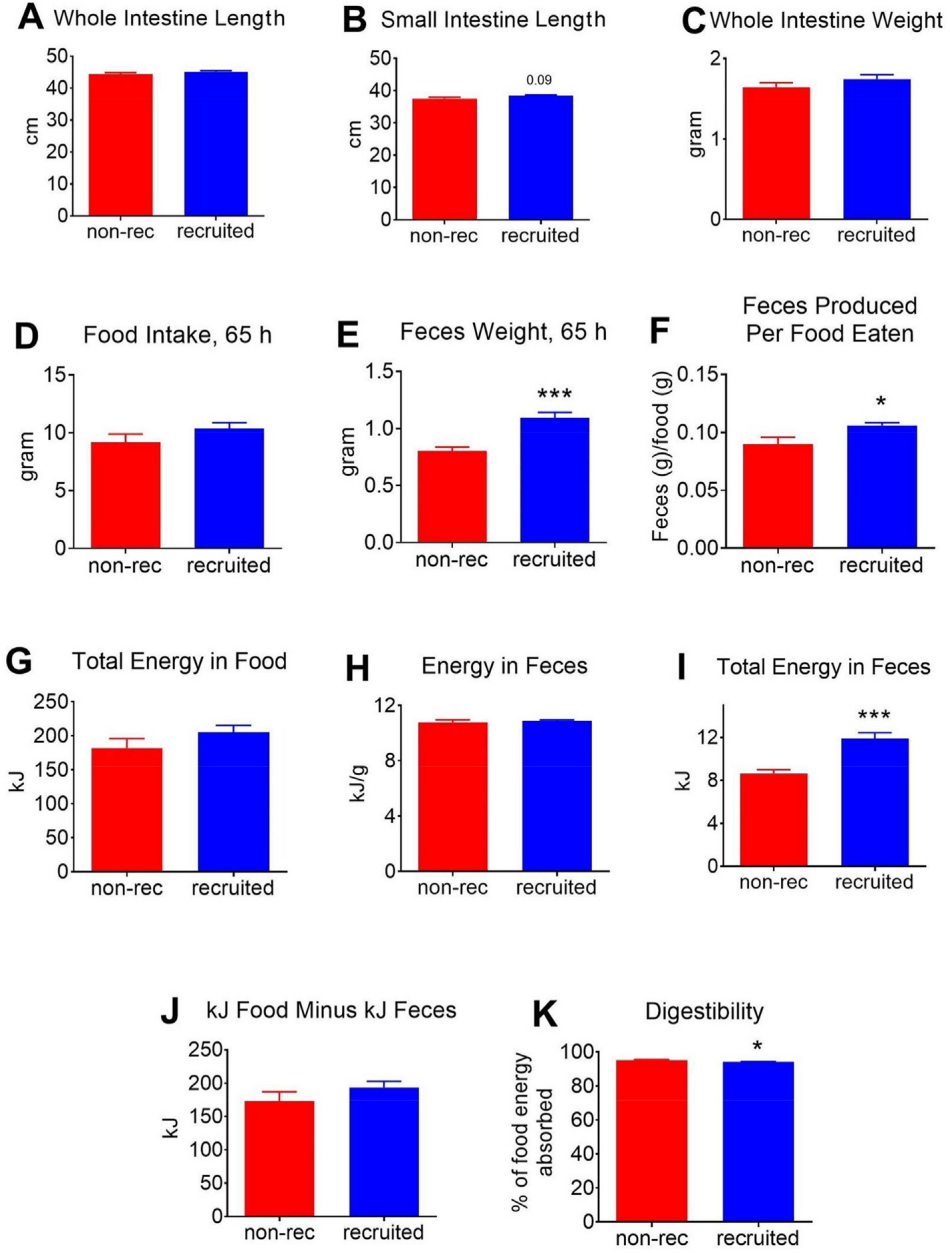
**Effects of BAT recruitment on food digestibility**. Another independent group of mice were treated as the mice in Figure 1, Figure 2. **A–C**: At the end of the experiment (day 18), the intestine was dissected out and measured in its total length (A) or only the small intestine (B), and the total intestine was weighed (C). **D–K**: Effects on digestibility. D: food intake during 65 h from day 4 in the afternoon to day 7 in the morning. **E**: total amount of feces collected during this period. **F**: ratio between the E and D. **G**: total energy in food eaten (based on the information from the producers). **H**: energy in feces (determined as detailed in Methods). **I**: total energy in feces (E multiplied by H). **J**: total energy absorbed (G minus I). **K**: digestibility (% of food ingested not excreted; ((G minus I)/I). n = 6 for all panels; significances as described in in legend to Figure 1.

Alternatively, the high efficiency could be caused by an improved ability to absorb food energy. We therefore examined the length and the weight of the intestines that could have been chronically increased during the recruitment phase, given that the recruited mice had to assimilate about one and a half times the amount of food during the recruitment process. However, there were no significant differences in these parameters (Figure 4A,B,C).

A similar possibility would be that the recruited mice, given their higher needs for energy, had in other ways acquired means to better digest the food. In independent cohorts, we therefore followed the food intake during the days (d4-d7) with very high metabolic efficiency, as well as the energy content of the feces during this time, and thus the digestibility of the food. In this cohort, the total food intake was slightly but not significantly higher during these days (Figure 4D). The feces weight was clearly higher in the recruited mice (Figure 4E) so on this basis the recruited mice demonstrated a lower, not a higher, food uptake efficiency (Figure 4F). As the energy content of the feces could have be changed and could thus mask an increase in efficiency, we determined the energy content in the feces, but this was not changed (Figure 4H). Thus, the recruited mice did not obtain the higher efficiency through an enhanced efficiency of food energy uptake; in reality the digestibility of the food in the recruited mice was slightly lower than in the non-recruited mice (Figure 4K).

### 3.7 The recruited mice deposit the extra energy with the theoretically maximal efficiency

As no change in metabolic rate or digestibility could be established, the question would be as to whether the rather modest increase in food intake during the initial days at thermoneutrality (Figure 2D) could be solely responsible for the body weight and body fat gain. During the first 11 days at thermoneutrality, the recruited group ate significantly more than the non-recruited group (Figure 2D). In total, the extra food intake during this time corresponded to 94 kJ (out of 665 kJ totally eaten by these mice after day 0), whereas the extra fat deposition corresponded to 86 kJ. Due to the cost of handling the food (“obligatory thermogenesis”), all energy digested cannot be converted into stored fat. As detailed in Supplementary Methods, using standard values for obligatory thermogenesis based on the composition of the diet used here, an expected obligatory thermogenesis of 8.7% can be calculated. Subtracting these 8.7% from the extra energy intake (94 kJ) yields an expected highest possible energy storage of 86 kJ, i.e. exactly the amount stored. Thus, the extra fat deposition could occur fully based on the extra food intake and does not require a concomitant reduction in metabolism or any other means.

### 3.8 Maintained thermogenic ability after transfer to thermoneutrality

The absence of any effect of the high amount of UCP1 on the metabolic parameters measured could reflect an inability of the UCP1 to be thermogenic. The mice had been transferred to thermoneutrality, and there would be no temperature-related demand on the system for generation of thermogenesis. Thus, an involution state of the tissue would arise, leading to the successive decrease in UCP1 content depicted in Figure 1. It might be suggested that during this involution process, the tissue may become unable to respond thermogenically to stimulation. We therefore examined the effect of β_3_-adrenergic stimulation on thermogenesis in the recruited and the non-recruited mice. As seen in Figure 3G, there was a large thermogenic response to CL-316,243 in the recruited mice when they were tested immediately after the end of the recruitment period; in the non-recruited mice, practically no response was seen. Importantly, when the mice were examined 4 days after cessation of the recruitment period (Figure 3H), the recruited mice still demonstrated a very marked thermogenic response. Thus, at the time when the mice demonstrated their highest fat deposition rate and the highest metabolic efficiency, the brown and brite/beige adipose tissues still possessed a large available thermogenic capacity. This capacity was, however, clearly not called upon metabolically under these conditions.

### 3.9 No qualitative or quantitative difference in metabolic efficiency due to the total absence of UCP1

The previous results implicated that the presence of exceedingly high UCP1 amounts did not lead in itself to decreased metabolic efficiency. To examine if any significance could be ascribed to UCP1, an additional series of experiments was designed, involving UCP1(−/−) mice, as well as wild-type mice. All mice were treated in the same way as in the previous studies, but food intake and body composition were measured every day (or as indicated). In the UCP1-ablated mice, the recruitment period can evidently not result in any augmentation in UCP1 levels, and the UCP1(−/−) mice that are exposed to the cold lack the ability to develop nonshivering thermogenesis and therefore shiver constantly and have markedly decreased longevity [19]. Additionally, the absence of UCP1 leads secondarily with time to alterations in mitochondrial structure and function, alterations that thus principally eliminate any effects of brown adipose tissue on oxidative metabolism [20,21].

**Figure 5:**
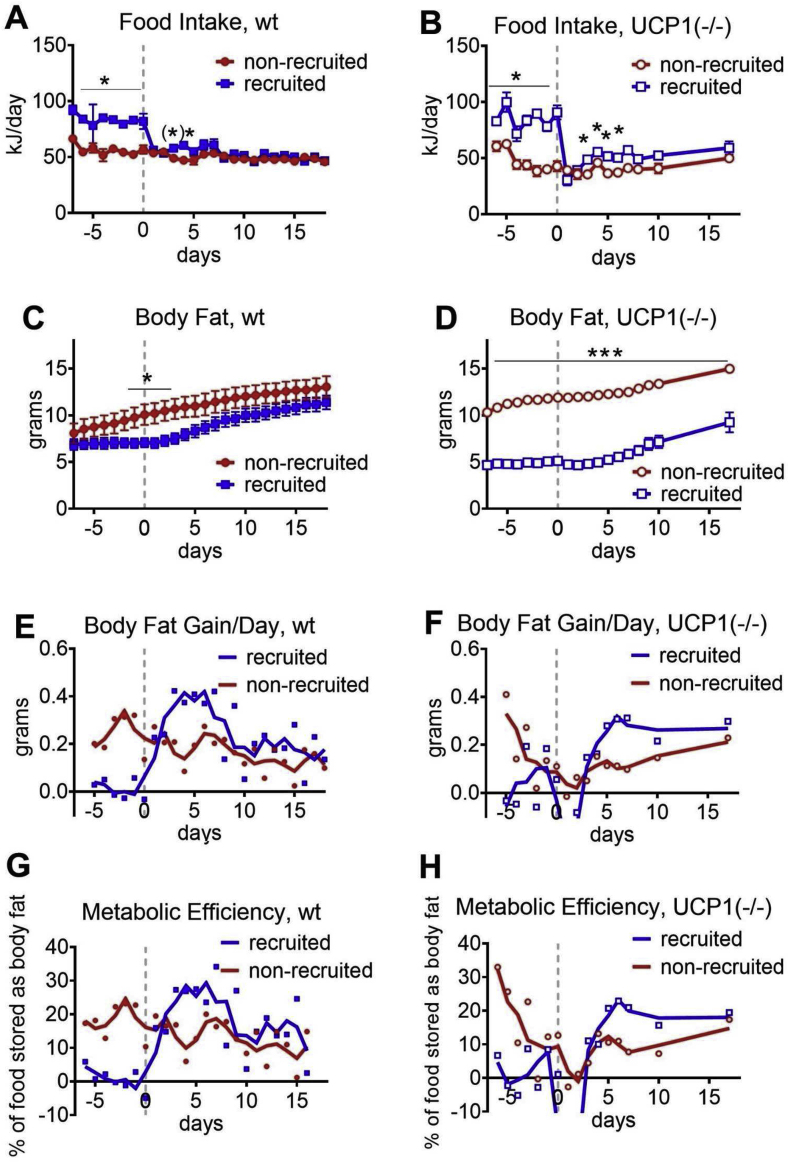
**No qualitative difference in metabolic efficiency between wildtype and UCP1-ablated mice**. A group of wild-type mice (ACEG) and a corresponding group of UCP1(−/−) mice (BDFH) were treated in the same way as in the previous studies, but food intake and body composition were measured every day; the two groups were not studied in parallel. Initial body weights at day −29 were for wild-type 28.1 ± 0.8 g for non-recruited and 27.7 ± 0.7 for recruited (means ± SE); for UCP1(−/−) 23.2 ± 0.8 for non-recruited and 23.0 ± 1.0 for recruited (means ± SE) (note that UCP1-ablated mice were smaller). **A:** Food intake in wild-type mice. **B:** Food intake in UCP1(−/−) mice. **C:** Body fat mass in wild-type mice. **D:** Body fat mass in UCP1(−/−) mice. **E:** Body fat gain per day in wild-type mice. **F:** Body fat gain per day in UCP1(−/−) mice. **G:** Metabolic efficiency in wild-type mice. **H:** Metabolic efficiency in UCP1(−/−) mice. In all panels, n = 5–8. Significances as described in legend to Figure 1. For E-H, the points represent the mean values of the single day measurements; the running curve is the smoothed curve calculated with 2 neighbours on each side.

Food intake in both wildtype and UCP1-ablated mice followed the same pattern as in the experiments above: it was stable in non-recruited mice and high in recruited mice at 4 °C but decreased immediately after the mice were transferred to thermoneutrality (Figure 5AB); this was followed, as before, by some days of slightly higher food intake than in the non-recruited mice. At the start of the experiment, the UCP1(−/−) mice at thermoneutrality had already gained somewhat more body fat than the wild-type (Figure 5CD). This is consistent with earlier studies that generally show that UCP1-ablated mice are more obesity-prone compared to wild-type mice when maintained at thermoneutrality and given high-fat diets [[22], [23], [24]], although this may not always be the case, as we have recently compilated [25].

In the wild-type mice, the transfer to thermoneutrality elicited exactly the same response as previously: the mice greatly increased the rate of body fat gain, exceeding that of the non-recruited mice, until the total amount of fat became approximately the same as that of the mice that had been kept at thermoneutrality; the rate then decreased to parallel that of the thermoneutral mice (Figure 5C,E). The “recruited” UCP1(−/−) mice had very low body fat amounts (Figure 5D); this is principally in accordance with [26,27] (and our unpubl. obs.), that showed that UCP1-ablated mice at subthermoneutral temperatures are obesity-resistant. Concerning body fat gain after transfer to thermoneutrality, the UCP1(−/−) mice showed qualitatively and quantitatively the same pattern as the UCP1(+/+) mice: an increase in the rate of gain, so that the rate exceeded that of the mice that had remained at thermoneutrality; thereafter a return to similar levels as in the non-recruited mice (Figure 5E,F), although within the time of the study, these mice did not reach the same body lipid amounts as the wildtype mice (Figure 5D). Metabolic efficiency followed the same pattern in the UCP1(−/−) mice as in the wild-type mice (Figure 5G,H).

Taken together, the recruited UCP1(−/−) mice did not have a qualitatively different response from recruited wild-type mice at thermoneutrality, indicating again that despite the presence of even excessively high amounts of UCP1 in wild-type mice, the protein is not at all active; indeed, it is as metabolically inactive as if it were not present at all.

### 3.10 Effects of brown adipose tissue recruitment on metabolic parameters

**Figure 6:**
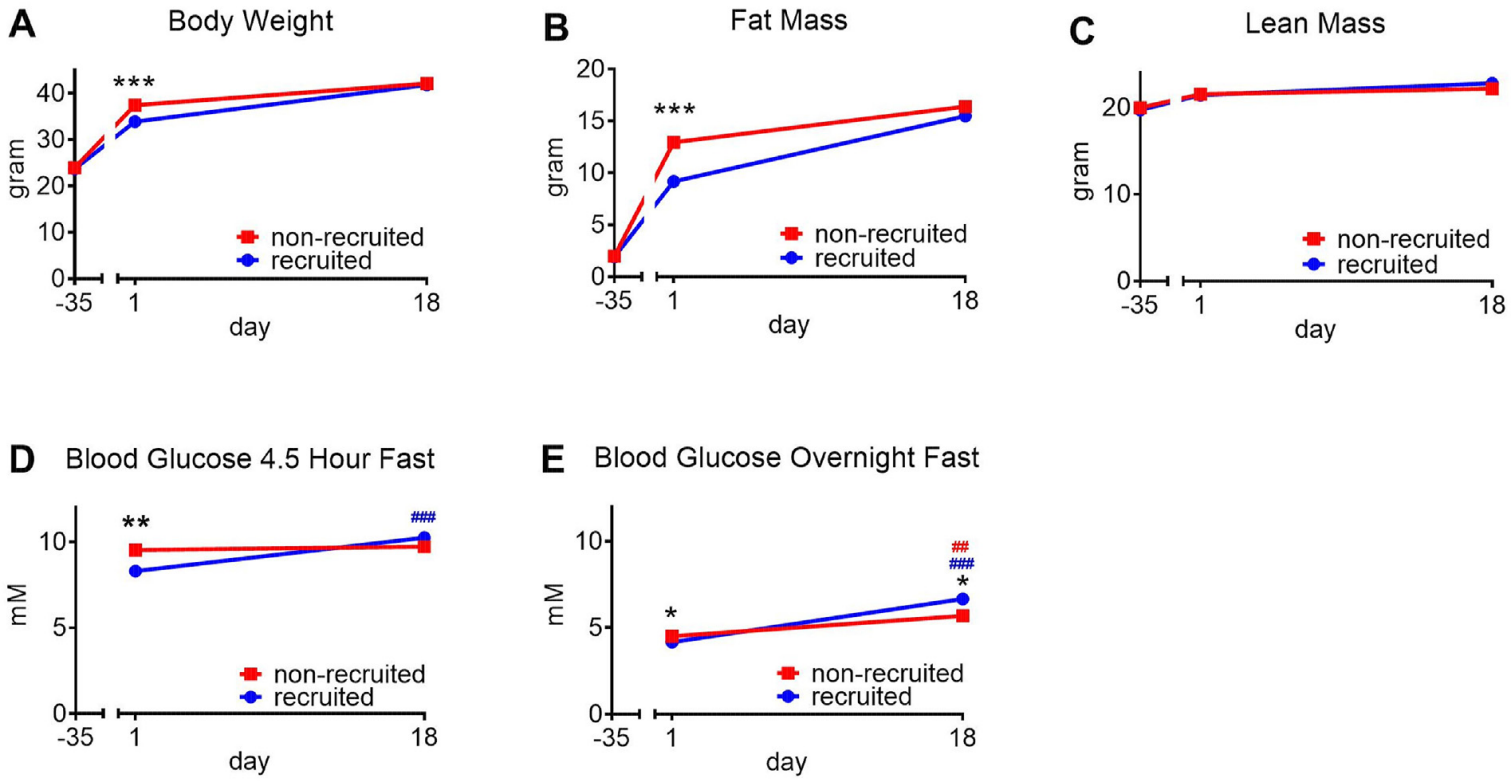
**Effect of BAT recruitment on blood glucose levels**. **ABC**: Independent groups of mice (the same as in Figure 4) were treated as those described in Figure 1 and their body weight, fat mass and lean mass followed; n = 18/18. **D**: On days 1 and 18 after transfer of the recruited mice to 29 °C, a group of mice were fasted for 4.5 h and their blood glucose levels determined as described in Methods; n = 12/12. **E**: On the same days, another group of mice were fasted overnight and their blood glucose levels determined; n = 6/6. Error bars were smaller than the size of the symbols. Statistics as in Figure 1. # etc. indicates significant differences between day 1 and day 18 (colour-coded). (For interpretation of the references to colour in this figure legend, the reader is referred to the Web version of this article.)

In an independent experimental series, we examined whether the recruitment process conveyed resistance to other metabolic effects of high-fat diet. The recruited mice in this series displayed again the remarkable re-adjustment to the fat mass and body weight of the non-recruited mice (Figure 6A,B,C).

With regard to the possibility that brown adipose tissue functions as a glucose sink and thus lower blood glucose levels [28], we found that after a 4.5 h fast, blood glucose levels were significantly lower in recruited mice than in non-recruited mice (Figure 6D). This difference had disappeared after 18 days. These observations would be in agreement with the recruited brown adipose tissue being involved in enhanced glucose disposal. This effect may not be due to UCP1 as such, since enhanced glucose uptake occurs even in stimulated UCP1-ablated mice [29,30]. Blood glucose levels after an overnight fast did not, however, show any biologically significant difference between recruited and non-recruited mice (Figure 6E).

**Figure 7:**
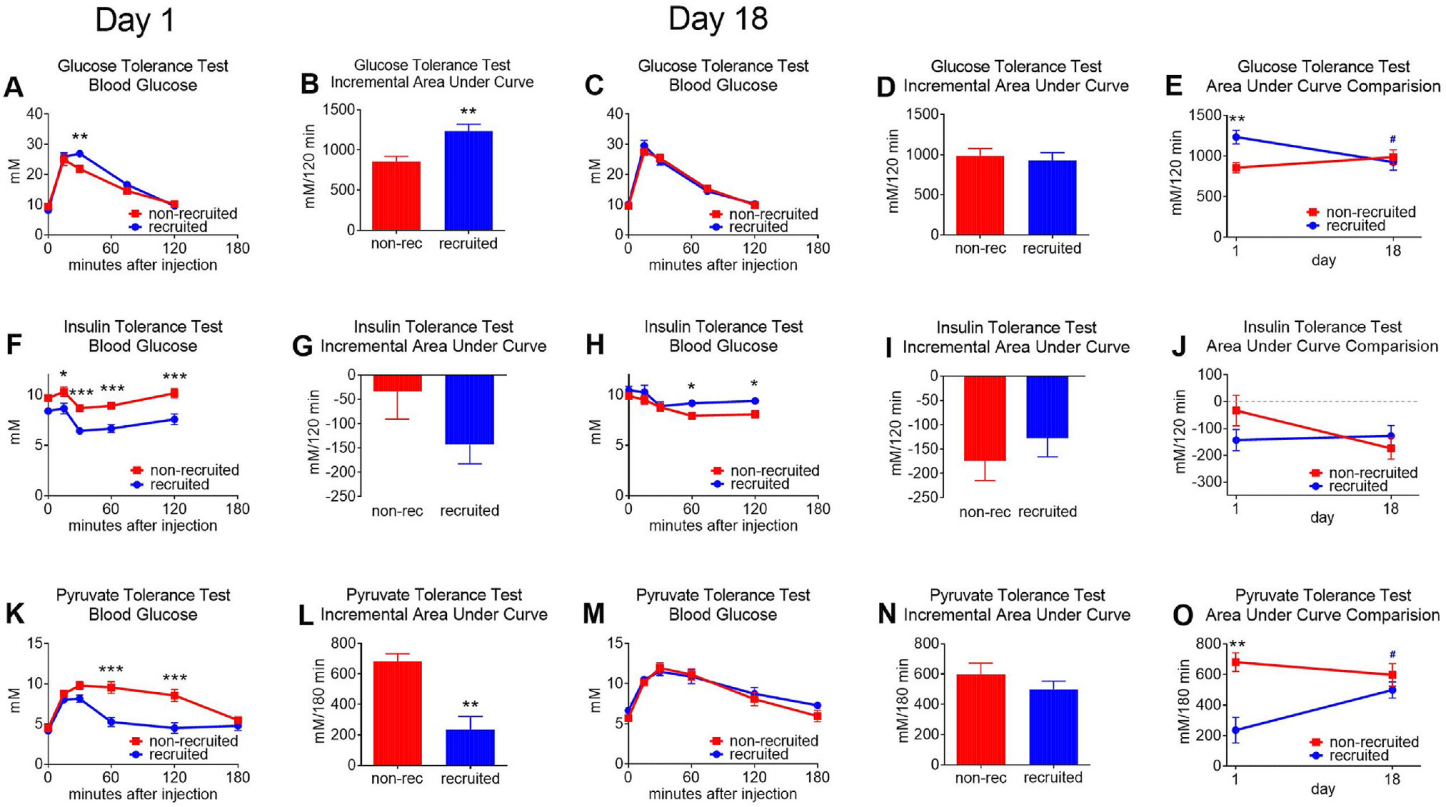
**Effect of BAT recruitment on glucose-related metabolic parameters**. The mice in Figure 4, Figure 7 were examined on the day they were transferred to thermoneutrality (AFK) and on day 18 after transfer (CHM). **A–E**: glucose tolerance tests. **F–J**: insulin tolerance tests. **K–O**: pyruvate tolerance tests. The compilation of the results in BDGILN are all incremental values, i.e. the initial blood glucose values were subtracted. Statistics as in Figure 1; # etc. indicates significant differences between day 1 and day 18 (colour-coded). (For interpretation of the references to colour in this figure legend, the reader is referred to the Web version of this article.)

In a glucose tolerance test (Figure 7A) (where we injected the same amount of glucose per g lean mass in all mice), the recruited mice on day 1 actually showed less glucose tolerance than the non-recruited mice. However, after 18 days at thermoneutrality, there was no longer any difference in the glucose tolerance test between the recruited and the non-recruited mice (Figure 7C,D,E). The initial lower glucose tolerance would tend to indicate that the innate insulin effects are lower in the recruited mice; this would mean that the low thermogenesis cannot be explained as being due to an inhibitory effect of insulin on thermogenesis – although insulin has this ability as such [31].

In the perhaps more relevant insulin tolerance test, although absolute glucose levels were lower, a non-significantly higher response to insulin was seen on day 1 in thermoneutrality (Figure 7F,G) but this tendency was not observed after 18 days (Figure 7H,I,J).

In a pyruvate tolerance test, a very marked higher “tolerance” was observed in the recruited mice (Figure 7K,L), implying that these mice were low in gluconeogenesis; this difference disappeared with time (Figure 7M,N,O).

Thus, the dramatically higher UCP1 levels in the recruited mice were only associated with minor alterations in metabolic parameters normally associated with a high-fat diet. Even the differences that are seen are only correlative, and the participation of UCP1 in the effects cannot be demonstrated from these results.

## 4 Discussion

In the present investigation, we have examined whether the acquisition of large amounts of UCP1 (in brown adipose tissue, as well as in brite/beige adipose tissue) in itself provides an augmented defense against diet-induced obesity and conveys an amelioration of other metabolic parameters. We found that despite extremely high levels of UCP1, at least 50 times more than in non-recruited control mice, these highly recruited mice transiently and unexpectedly gained *more* weight and body fat than controls when examined under “physiologically humanized” [32] conditions. The results are of significance for the understanding of body weight control as such, and of browning, and for the development of new drugs and pathways to counteract the development of obesity.

### 4.1 The effect of pre-recruitment on energy balance

The outcome of the present experiments can be described as follows. The control mice were exposed throughout the experiment to conditions (high-fat/high sugar diet and thermoneutrality) that lead to induction of diet-induced thermogenesis, characterized by a small but metabolically significant increase in UCP1 amounts and increased energy expenditure during feeding (as compared to chow-fed mice), as earlier shown [8,22], processes that are probably mediated by the sympathetic nervous system [33]. Still, despite this thermogenesis, the mice steadily increase their lipid stores, indicating that the level of diet-induced thermogenesis induced here cannot fully compensate for their high energy intake.

The “recruited” mice should be in a different situation. They have been exposed to the highly recruiting conditions of cold exposure and have acquired large amounts of UCP1 and metabolically expanded brown adipose tissue. When they are then transferred to thermoneutrality, they are still exposed to the high-fat/high sugar diet that should activate diet-induced thermogenesis via stimulated sympathetic innervation. However, in contrast to the controls, they already have access to the vast capacity for energy expenditure found in their recruited brown adipose tissue and they should be able to activate this through the sympathetic nervous system and thus counteract obesity development to a greater extent. This is, however, a possibility that is not utilized. Instead, they start to overeat, and, despite their access to their highly recruited brown adipose tissue, they channel all the available energy of the extra food into their lipid stores. Thus, to replenish these stores and bring the reserves to exactly the level found in the control mice overruns the possibility to use the brown adipose tissue for enhanced diet-induced thermogenesis, leading to the mice actually becoming obese at a higher rate than the controls, to finally arrive at exactly the same state of obesity as the controls.

Thus, the mere presence of high amounts of UCP1 and highly recruited brown and brite/beige adipose tissues does not ensure protection against obesity, and the obese state observed in high-fat/high-sugar-fed mice should not be understood as the passive outcome of overeating but as a condition actively strived for by the metabolic control systems of the mice under these exact conditions.

### 4.2 The thermogenic capacity of large amounts of UCP1 is not obligatorily utilized to protect against obesity

Although much effort is presently directed towards augmentation of UCP1 amounts in both brown and brite/beige adipose tissues (see Section 1), there are no direct studies demonstrating the effects of increased UCP1 amounts as such. For instance, although many “browning agents” have been shown to increase UCP1 in e.g. inguinal WAT *and* to lead to lower fat mass, it has not been investigated whether the fat-lowering effect is dependent upon the increase in UCP1 amount – or whether the increase in ingWAT UCP1 is a consequence of the reduction of fat mass through increased sympathetic stimulation, rather than being the cause of it.

Principally, true “browning agents” (as distinguished from the “false-positive” browning agents that act indirectly by augmenting heat loss [2]) may be of two types: those that mimic or augment some step in the adrenergic/cAMP cascade that thus leads to both enhanced cell differentiation (increased UCP1 levels) *and* to acute activation of UCP1 – or those that only increase differentiation and not thermogenesis, working through other pathways (e.g. PPARγ activators [[34], [35], [36], [37]]). These latter effectors may be subdivided into those that directly act on UCP1 gene expression and thus increase UCP1 amounts without any general augmentation of the thermogenic capacity of the tissues – and those that also induce mitochondriogenesis, vascularization etc.

Here, we examined the effect of a recruited tissue with an augmented amount of UCP1 under conditions when the acute stimulation from cold had been removed. Thus, the entire thermogenic system, not only the amount of UCP1, would be enhanced and would be available for thermogenesis induced by any other means than cold. Here, the recruited tissue should be stimulated by the high-fat diet used, earlier demonstrated to induce UCP1-dependent diet-induced thermogenesis [8]. We found that although the mice possessed very large amounts of UCP1, this UCP1 was not utilized by the mice and it did not in itself lead to enhanced thermogenesis. Indeed, the mice could control their metabolism to the extent that they at least transiently became even *more* metabolically efficient than the mice with low amounts of UCP1 and therefore gained more body fat. Thus, augmenting the total UCP1 content, even when this is done physiologically so that the total thermogenic machinery is recruited, does not in itself affect energy metabolism.

### 4.3 UCP1 is not leaky

The results presented here also have bearing on the tenet that UCP1 is not leaky. Both when studies are performed in isolated brown-fat mitochondria [38] or in isolated brown-fat cells [39], the conclusion is that when UCP1 is not in an active state, there is no observable uncoupling effect of the presence of UCP1; nor is there any effect of the presence or absence of UCP1 on basal metabolic rate in animals (mice) [40]. However, in the present experiment, the conditions are different: the diet used here has been demonstrated to induce UCP1-dependent diet-induced thermogenesis [8], and therefore the diet should generate signals that should be able to activate the large amounts of UCP1 present. That this does not occur implies that the central control of the sympathetic stimulation of brown and brite/beige adipose tissues is more intricate than is presently understood.

### 4.4 A “variable” adipostat

A general notion is that most adult animals, including humans, tend to keep a relatively constant body weight: the balance between food energy intake and that combusted is generally better than 99%. This is generally discussed as indicating the existence of a mechanism, referred to as an adipostat or a lipostat. Functionally, this is ascribed to the brain being informed of the status of the body's energy reserves and thus taking adequate countermeasures if body weight/body energy reserves deviate from desired levels [[41], [42], [43], [44], [45]]. Leptin is generally thought to be the (main) conveyer of this information from the periphery to the brain [46], but there are indications that other systems may be involved [[47], [48], [49], [50]]. The general question as to whether such a system for body weight control really exists, with a set point or with settling points, and how it could develop, has been discussed, based on e.g. the outcome of mathematical models [51,52].

Serendipitously, our observations here at least at first glance support the existence of such an adipostat. The “recruited” mice gained just sufficient amounts of fat to reach the same fat content as those mice of the same age that had been living at thermoneutrality for the duration of the experiment (Figures 2A,B, Figure 5, Figure 6B). The increase in body weight (obesity) seen e.g. in the non-recruited mice was thus not the passive result of a marginal excess of food intake; rather, it would seem that it was a “goal” set by the given conditions. However, the mice would not seem to have one fixed and specified fat set point; rather, given a series of conditions, including environmental temperature, the nature of the food, the age, the gender, the mice will strive to reach a very specified weight/fat content. This thus indicates that such body weight changes are not the result of haphazard alterations in energy balance but appear programmed in the organism.

### 4.5 The acute regulation of thermogenesis

It seems that the brain strives to obtain a given accumulation of energy reserves in the body and this goal overrules other types of signals. High amounts of fat in the body have been suggested to activate thermogenesis to counteract further obesity, and leptin has been assumed to mediate this signal [53] (although leptin is actually not thermogenic [54,55]). However, the brain clearly does not oppose obesity as such, and based on the data presented here, it prevents the occurrence of diet-induced thermogenesis until a predetermined degree of obesity has been reached. A high food intake as such may also be associated with (obligatory) diet-induced thermogenesis, but in the mice studied here, the period with the highest food intake at thermoneutrality coincided with the period of time when metabolic efficiency was at its highest (Figure 2D,E).

### 4.6 The interaction between diet-induced thermogenesis and cold-induced thermogenesis

The experiments described here were all conducted at thermoneutrality (≈30 °C). In our opinion, this is the only condition under which facultative diet-induced thermogenesis can be observed. As earlier discussed by us and others [2,56], at environmental temperatures below thermoneutrality, any increase in energy expenditure caused by any agent will be used as part of the thermoregulatory thermogenesis that is necessary for compensating the heat loss, to maintain the body temperature. This is experimentally observed as apparent elimination of the thermogenic effect of artificial uncouplers, as well as of the thermogenic effect of physical activity [[56], [57], [58]]. Thus, if the present experiment had been conducted at standard housing temperatures (≈20 °C), it would not even theoretically have been possible to observe an augmented diet-induced thermogenesis in the recruited mice. Correspondingly, that such thermogenesis was not observable under the present conditions demonstrates that the mice can acutely regulate the fraction of the thermogenic capacity present that is actually used for diet-induced versus cold-induced thermogenesis.

As we humans spend most of our time under thermoneutral conditions [59,60], any facultative diet-induced thermogenesis that exists should be manifest in humans.

### 4.7 Are the attempts to identify pathways for augmenting UCP1 amounts in order to counteract obesity doomed to be in vain?

In many studies aimed at increasing thermogenesis with the goal of counteracting the development of obesity, the stated successful endpoint reached has often been increased UCP1 amounts or increased UCP1 gene expression levels. The present study demonstrates that even if such an endpoint is achieved, the augmented amounts of UCP1 can be without anti-obesity function. The challenge pharmaceutically and nutraceutically is thus not only to enhance the total amount of UCP1 in human subjects but also to ascertain that the UCP1 is adequately activated. If this is accomplished, the induced thermogenesis could decrease metabolic efficiency and thus curtail the development of obesity.

## Author contributions

GvE, BC and JN devised the study; GvE, PZ, EM and EL performed the experiments, GvE and JN wrote the manuscript, and all authors revised and approved the manuscript.

## Data Availability

Data will be made available on request.
