# Supplementary material for: Highly recruited brown adipose tissue does not in itself protect against obesity
Source: Mol Metab. 2023 Jul 25;76:101782. doi: 10.1016/j.molmet.2023.101782 (PMC10432997; doi:10.1016/j.molmet.2023.101782)
Supplement: Multimedia component 1 [file mmc1.docx]

**Supplement:**

**Detailed experimental procedures**

**Animals**

All experiments were approved by the North Stockholm Animal Ethics Committee. Wild-type male C57Bl/6 mice (8–12 weeks old), obtained from Scanbur (Europe), were used in all experiments described below. In addition, male UCP1(-/-) mice were used in experiment 3. These UCP1(-/-) mice were from our own breeding, descendants of the mice described in [1] and backcrossed to the C57BL/6 strain for more than ten generations. These mice were kept as homozygous breeding lines, backcrossed (and then outcrossed) to wildtype C57Bl/6 every 10^th^ generation.

In all experiments, the mice were exposed to a light-dark 12/12 h cycle, were single-caged and had *ad libitum* access to food and water. Before the studies started, all the mice were kept on chow (R70, Lactamin) at 22 °C. In all experiments, half of the mice were pre-exposed to cold (4 °C) for about a month (day -33) to induce a very high recruitment of their brown and brite/beige adipose tissues; the other half were placed in principally thermoneutral temperature (29 °C). At the start of the experiment (day 0), the mice in the cold were transferred to thermoneutrality. From day -33, all mice were fed high-fat diet (see below) *ad libitum*. The effect on metabolic parameters, especially on body fat acquisition, of the recruitment of brown and brite/beige adipose tissues by the cold acclimation was then studied, as detailed below.

*Experiment 1*: The study was initiated when the mice were 12 weeks old, indicated as day -33, by killing 5 mice for tissue analysis. The remaining mice were switched to a high-fat diet and placed at one of the different ambient temperatures (4 °C or 29 °C, referred to as “recruited” or “non-recruited” mice). After 33 days, 5 mice at each temperature were killed and the remaining mice at 4 °C were transferred to 29 °C (indicated as day 0). Food intake was measured from day -17 to day 18. Weight and body composition were obtained as indicated. At the indicated time-points (day 0, day 4 and day 18) 5 recruited mice and 5 non-recruited mice were killed, and tissues were taken and further analyzed as described below.

*Experiment 2*: A group of 12 mice (the same background and age as in exp. 1) were treated the same way as in exp. 1. However, at day 0, all the mice were directly transferred to metabolic chambers held at 29 °C and were followed therein from day 0 to day 3.

*Experiment 3:* Wild-type and UCP1(-/-) mice were kept under principally the same conditions and treatments as mice in exp. 1, but measurements of food intake and body composition were performed more often, as indicated. The UCP1(-/-) mice were housed at 18 °C for 12 days before transfer to 4 °C for another 21 days [2].

*Experiment 4*. 12 wildtype mice were treated as in exp 1. On day 1 and day 4 after transfer, the mice were examined for thermogenic capacity, as described below.

*Experiment 5.* 36 wildtype mice were treated as in exp. 1. At day -35, day 0 and day 17, the mice were weighed and body composition examined. On day 1 after transfer to thermoneutrality, 12 of the mice (6 recruited and 6 non-recruited) were examined with a glucose tolerance test, 12 mice with an insulin tolerance test, and 12 with a pyruvate tolerance test, as described below. From day 4 in the afternoon until day 7 in the morning (3 nights, 65 h), total food intake was monitored and all feces was collected in 6 recruited and 6 non-recruited mice. The energy content of feces was determined as described below. On day 18, the tolerance tests as indicated above were again performed.

**Diets**

From day -33, all mice were fed a high-fat diet. For calculations of the energy content and the composition of the diet, we used the values specified by the manufacturer of the food. The high-fat diet (D12451, Research Diets, New Brunswick), was semisynthetic and contained 45 energy% fat, 20 energy% protein and 35 energy% carbohydrates (of which 17 energy% fructose), with a total energy content of 19.8 kJ/gram. Based on respiratory exchange rates (RER) of 0.70 for fat and 1.00 for protein and carbohydrates, the theoretical RER of this high-fat diet is 0.84. Based on calculations using standard thermic effects of fat (2.5 %), carbohydrates (7.5 %) and protein (25 %) (Food and Nutrition Board, 2005), the theoretical obligatory diet-induced thermogenesis (“thermic effect”) of the high-fat food is 8.7 %.

**Body composition and metabolic efficiency**

Body fat content and lean body mass of mice were measured by *in vivo* magnetic resonance imaging (MRI) using EchoMRI™ Whole Body Composition Analyzer at the indicated time-points. The analyzes yielded total body fat content and lean body mass. Metabolic efficiency, expressed as percent of energy intake stored as body fat, was calculated as the body fat gain (in grams) for the relevant period, multiplied by 39 kJ/g and divided by kJ food intake for the same period.

**Energy expenditure and respiratory exchange ratio**

To determine energy expenditure, oxygen consumption and carbon dioxide production were measured by indirect calorimetry (INCA Systems, Somedic, Hörby, Sweden). The mice in exp. 2 were placed in their home-cages in the metabolic chambers at 30 °C for two days and three nights. Respiratory exchange ratios were calculated by dividing V_CO2_ with V_O2_. To estimate energy expenditure, the Weir equation was used: (16.3 J/ml x ml/min O_2_ used) + (4.6 J/ml x ml/min CO_2_ produced) [3], yielding J/min and recalculated to watt by dividing by 60.

In order to examine thermogenic capacity, the mice in exp. 4 were placed in the metabolic chambers at 30 °C and injected with 1 mg/kg bw of CL-316,243, and the rate of oxygen consumption was followed for 6 hours.

**Protein analysis**

After sacrificing the animals from exp. 1 with carbon dioxide and cervical dislocation, the entire depots of interscapular brown adipose tissue and inguinal white adipose tissue were dissected out and weighed. The tissues were immediately frozen in liquid nitrogen and stored at -80 °C. For protein analysis, tissues were homogenized in RIPA buffer (50 mM Tris-HCl pH 7.4, 1 % Triton X-100, 150 mM NaCl and 1 mM EDTA) with 1 tablet of protease inhibitor cocktail (Complete-Mini, Roche Diagnostics) per 10 ml of buffer. In addition, 5 mM NaF (serine/threonine phosphatase inhibitor) and 1 mM Na_3_VO_4_ (tyrosine phosphatase inhibitor) were added to the buffer. The buffer was added to samples in volumes of 10 ml/g IBAT and 3 ml/g ingWAT. The tissues were homogenized, lysed on ice for 15 min and centrifuged at 14,000 *g* for 15 min, and the aqueous intermediate phase was collected. To determine total protein concentration, the Lowry protein method and the Pierce™ BCA Protein Assay Kit were used.

**Western Blotting**

For determination of UCP1 protein levels, protein (2 µg protein for IBAT (and for day 0 in recruited ingWAT) and 10 µg protein for the other ingWAT samples) were loaded on an SDS-polyacrylamide gel. Technical validation experiments indicated that higher amounts of protein per lane easily resulted in saturation of the signal and thus to underestimation of total UCP1 amounts. To be able to quantify and compare different membranes, a standard sample was loaded on each gel (the standard sample consisted of pooled brown adipose depots previously aliquoted in small tubes and prepared in the same way as the samples).

The amount of UCP1 in the standard sample was set to 1.00 AU on each gel. For IBAT, 1.00 AU, and for ingWAT an amount corresponding to 0.15 AU, of the standard sample was loaded on each gel. In addition to the ingWAT samples, the ingWAT-gels were also loaded with a sample of protein from BAT of UCP1(-/-) mice. After electrophoresis, proteins were transferred by electroblotting to a polyvinylidene difluoride membrane. UCP1 protein was determined by incubation with UCP1 polyclonal antibodies (prepared in rabbit from the C-terminal decapeptide of mouse UCP1) as primary antibody and Anti-Rabbit IgG HRP-linked antibody (Cell Signaling) as secondary antibody. Dilution of both antibodies was 1:12 000. The membranes were incubated with detection reagent (Clarity Western ECL Substrate, BioRad), and the chemiluminescence signal was detected with a CCD camera (Fuji). Quantifications were performed with Image Gauge 3 software.

Traditional loading controls in a highly plastic tissue such as brown adipose tissue are not presently identified and may not exist, i.e. no single protein has been demonstrated to keep the same level per mg tissue protein in the different adaptive states of the tissue. For general confirmation of the same protein loading, Ponceau staining was routinely performed.

**Tolerance tests**

For glucose tolerance tests, mice were fasted 5.5 h from 9:30 and then injected i.p. with 3 mg/g lean body mass of D-glucose (corresponding approx. to 2 g/kg body weight). Before the glucose injection and in regular intervals after it (see corresponding graphs), blood glucose was measured in a small drop of blood from the tip of the tail using a glucometer AccuChek Aviva.

For insulin tolerance tests, mice were fasted 4.5 h from 9:30 and then injected i.p. with 1.65 IU/kg lean body mass of insulin (corresponding approx. to 1 IU/kg body weight). Blood glucose was determined as for the glucose tolerance test.

For pyruvate tolerance tests, mice were fasted 14 h from 7 pm the previous day and then injected i.p. with 3 mg/g lean body mass of pyruvate (corresponding approx. to 2 g/kg body weight). Blood glucose was determined as for the glucose tolerance test.

**Feces energy content**

Energy content was determined with a high temperature differential scanning calorimetric system (NETZSCH STA 449F3 Jupiter system). Samples were homogenized with a FastPrep 24® for 2 min. The temperature was scanned at 10 °C/min from room temperature to 900 °C. Total enthalpy of combustion was measured as the area under the curve.

**Statistical analyzes**

The results were analyzed in MS Excel. All data are expressed as means ± standard error (SE). Unpaired Student’s t-test with unequal variances was applied to calculate significance. Significance is indicated when recruited differed from non-recruited, as (*) for p-values ≤ 0.1, * for p ≤ 0.05, ** for p ≤ 0.01 and *** for p ≤ 0.001. In tolerance tests, # is similarly used to indicate differences between day 1 and day 18 values.

**References**

[1] S. Enerbäck, A. Jacobsson, E.M. Simpson, C. Guerra, H. Yamashita, M.-E. Harper, L.P. Kozak, Mice lacking mitochondrial uncoupling protein are cold-sensitive but not obese, Nature 387 (1997) 90-94.

[2] V. Golozoubova, H. Gullberg, A. Matthias, B. Cannon, B. Vennstrom, J. Nedergaard, Depressed thermogenesis but competent brown adipose tissue recruitment in mice devoid of all hormone-binding thyroid hormone receptors, Molecular endocrinology 18(2) (2004) 384-401.

[3] J.B. Weir, New methods for calculating metabolic rate with special reference to protein metabolism, J Physiol 109(1-2) (1949) 1-9.
