# Supplementary material for: Highly recruited brown adipose tissue does not in itself protect against obesity
Source: Mol Metab. 2023 Jul 25;76:101782. doi: 10.1016/j.molmet.2023.101782 (PMC10432997; doi:10.1016/j.molmet.2023.101782)
Supplement: Multimedia component 2 [file mmc2.docx]

**A. Relationship between body weight and energy expenditure**

**B. Relationship between body weight and food intake**

**C. Relationship between food intake and energy expenditure**

**Legend to supplementary figure**

The data underlying Fig. 3B and 3C are plotted here, principally for ANCOVA analysis, with the energy expenditure (A) and the food intake (B) as functions of body weight. The data do not statistically correlate with body weight.

In C, energy expenditure is plotted as a function of food intake.
